# Impact of sterile *Aedes aegypti* males releases on vector dynamics: insights from Malaysian field trials

**DOI:** 10.1186/s40249-025-01303-x

**Published:** 2025-04-30

**Authors:** Wasi Ahmad Nazni, Guat-Ney Teoh, Mohd Adnan Nuradila, Shaikh Ismail Shaikh Norman Hakimi, Maheswaran Tanusshni, Mohd Azam Muhammad Arif, Achim Nurfarahin Hanini, Irfan Ahmad Shazia, Aik-Meng Tan, Hamzah Rabizah, Mohamad Dzomir Ahmad Zainuri, Asim Hasnor Hadi, Ahmad Norazah, Han-Lim Lee, Hamidou Maiga, Jeremy Bouyer, Yoon-Ling Cheong

**Affiliations:** 1 Medical Entomology Unit, Infectious Disease Research Centre, Institute for Medical Research (IMR), National Institutes of Health, Ministry of Health Malaysia, Jalan Pahang, 50588 Kuala Lumpur, Malaysia; 2 Entomology and Pest Unit, Malacca State Health Department, Malacca, Malaysia; 3 Division of Agrotechnology and Biosciences, Malaysian Nuclear Agency, Bangi, Kajang, Selangor Malaysia; 4 Health Education Unit, Malacca State Health Department, Malacca, Malaysia; 5 Biomedical Museum Unit, Special Resource Centre, Institute for Medical Research, National Institutes of Health, Ministry of Health Malaysia, 50588 Kuala Lumpur, Malaysia; 6 Direction Régionale de L’Ouest, (IRSS-DRO), Institut de Recherche en Sciences de La Santé, Bobo-Dioulasso, 01, 01 BP 545, Bobo-Dioulasso, Burkina Faso; 7 Insect Pest Control Subprogram, Joint FAO/IAEA Centre of Nuclear Techniques in Food and Agriculture, Vienna, Austria; 8 ASTRE, CIRAD, INRAE, Technological Platform CYROI, University of Montpellier, Sainte-Clotilde, La Réunion, France

**Keywords:** Sterile insect technique, Optimum dosage, Mean larval density, Mark-release-recapture, Spatial interpolation

## Abstract

**Background:**

The Sterile insect technique (SIT) has been successfully used in agricultural pest control, leading to interest in its application for public health, particularly in controlling *Aedes* mosquitoes in the USA, Italy, Cuba, and Greece. Malaysia has conducted a small-scale SIT pilot trial since 2019 for dengue control. This study evaluates mosquito populations in treated and untreated sites through three objectives: (1) comparing mean larvae per trap (MLT) and dengue cases for *Ae. aegypti* and *Aedes albopictus*; (2) estimating survival rates and wild populations using mark-release-recapture (MRR); and (3) analysing spatial distribution in treated and untreated sites.

**Methods:**

*Ae. aegypti* males, irradiated at 55 Gray, were released in three locations: Pangsapuri Kota Laksamana (KT), Malacca (19 months), Pangsapuri Taman Tasik Utama (TTU), Malacca (8 months), and the Customs, Immigration, and Quarantine Complex (CIQ), Johor (7 months). Statistical analyses assessed SIT effectiveness, including T-tests for larval density and ovitrap indices, Mulla’s formula and relative variance (RV) for population reduction, and the Lincoln Index for estimating wild male populations and probability of daily survival.

**Results:**

Weekly releases of sterile *Ae. aegypti* males at doses of 1278–7942 males/ha achieved a sterile-to-wild male ratio of 5.85 and a mean daily survival rate of 0.61, leading to significant reductions in larval densities: 76.25% in Kota Laksamana (KT), 96.74% in Taman Tasik Utama (TTU), and 89.00% in CIQ Gelang Patah, thereby supporting dengue control efforts. In KT, the MLT was initially low but increased, although with suppression < 90%, there was a reduce of dengue cases throughout the release period. The MRR’s mean survival rate (± standard deviation) in KT was 0.61 (± 0.08). The spatial clustering of *Ae. aegypti* was observed in central blocks during the high MLT period. However, larval densities rebounded after releases ceased. Spatial clustering revealed no initial clustering, though clustering patterns emerged over time in KT.

**Conclusions:**

SIT effectively suppressed *Ae*. *aegypti* populations and supported dengue control. Optimizing sterile-to-wild male ratios, spatial distribution, and monitoring strategies is essential for sustainable vector control. These findings provide insights for scaling up SIT field trials, with future efforts focusing on refining release and monitoring strategies to enhance SIT as an effective dengue control tool.

*Trial registration* NMRR-17–2652-39,099 “Field evaluation of Sterile Insect for *Aedes aegypti* Suppression.”

## Background

The introduction of *Aedes aegypti* (Linnaeus, 1762) to Malaysia from tropical Africa via India in the early twentieth century [1] marked a significant event in the country's entomological history. While *Aedes albopictus (Ae. albopictus)* (Skuse, 1894) is native to Southeast Asia, including Malaysia, both species have become globally important due to their roles as vectors for various human arboviruses. These mosquitoes are responsible for transmitting diseases such as dengue, Zika, and chikungunya, with *Ae. aegypti* being the primary vector for dengue fever [2]. *Aedes* mosquitoes, especially *Ae. aegypti*, are notorious for their ability to breed prolifically in artificial containers, making them well-adapted to urban environments.

Dengue outbreaks in Malaysia, as in many other parts of the world, have reached epidemic proportions in recent years, posing significant challenges to public health systems. Recent dengue outbreaks have been attributed to a combination of rapid urbanization, increased international travel, climate change, and underlying socioeconomic factors [3–5]. Dengue cases reported to the WHO have surged eightfold since 2000, from 505,430 to over 2.4 million in 2010 and 5.2 million in 2019. At 70% of the global tropical and subtropical illness burden, Asia is most afflicted [3, 4]. Malaysia reported 90,304 cases and 1452 deaths in 2020. There were 66,102 cases and 56 deaths by 2022 [6]. Dengue cases reached 122,423 in 2024, killing 117 [7]. These outbreaks result in substantial morbidity and mortality, highlighting the necessity for effective vector control measures. Developing effective solutions to prevent the spread of arboviruses transmitted by *Aedes* mosquitoes necessitates a thorough understanding of their ecology, behaviour, and geographical distribution. In the absence of specific antiviral treatments, effective tetravalent vaccines, preventive measures, or therapeutic agents, the elimination of dengue vectors becomes critical. The current national dengue control strategy in Malaysia emphasizes targeting mosquito vectors through methods such as adulticiding, larvaciding, and source reduction. The Ministry of Health's Vector Control Unit conducts larvaciding more frequently than adulticiding, employing temephos and *Bacillus thuringiensis israelensis* (Bti) as preventive measures and during outbreaks [8]. However, given the persistent challenges in effectively controlling dengue transmission, there is a clear need for innovative strategies, such as the sterile insect technique (SIT), to enhance vector management and reduce mosquito populations more sustainably.

The sterile insect technique, conceived by Edward F. Knipling in the 1950s, was initially used to control the New World screwworm (*Cochliomyia hominivorax*). A 45–year area-wide campaign achieving suppression and eradication in the USA, Mexico, Central America, some Caribbean islands, and North Africa (Libya) [9]. SIT has a long track record of success against a range of agricultural pest insects in fruit flies and Lepidoptera [10–12], and these success had led to increase in the use of SIT against public health pests particularly *Aedes* mosquitoes, which spread diseases like dengue, chikungunya, and Zika [13–15]. Early SIT trials targeting *Ae. aegypti* in the USA were inconclusive due to a lack of understanding of sterile males' mating competitiveness [16]. However, over the past two decades, the SIT strategy has been primarily focused on controlling *Aedes* species, specifically *Ae. aegypti* and *Ae. albopictus* trials on *Ae. aegypti* and *Ae. albopictus,* owing to their involvement *in* transmitting diseases like dengue, chikungunya and zika [17–25]. These efforts have shown significant success in reducing mosquito populations, including trials in Italy, Greece, and Cuba [15, 24, 25].

While SIT has demonstrated effectiveness in controlling *Aedes* mosquitoes, studies on its impact on other species like *Culex* and *Anopheles* mosquitoes vectors for diseases such as West Nile virus, filariasis, and malaria are limited. Some research has shown potential in using SIT for *Anopheles arabiensis* mosquitoes, but further studies are needed to understand its feasibility and to develop strategies for controlling these species effectively [17, 19, 21, 26, 27].

In light of these challenges, novel and innovative methods such as SIT has been developed to control the vectors in a phased conditional approach (PCA) meaning advancement to the next phase depends on completion of activities in the current phase [28]. The SIT was among the first biological insect control methods designed for areawide application (AW-A) [29].

The study’s specific objectives centred on analysing the field population of mosquitoes in treated areas using *Ae. aegypti* sterilized with gamma irradiation (Cesium-137) and comparing them with wild *Ae. aegypti* populations from untreated sites. We first analysed and compared the mean larval density per trap for both *Ae. aegypti* and *Ae. albopictus*, along with dengue case data, between treated and untreated sites. Next, we conducted mark-release-recapture (MRR) studies to estimate the survival rates and wild population sizes of *Ae. aegypti* in release areas. Additionally, spatial interpolation was used to compare the field population dynamics between treated and untreated sites, providing insights into the spatial distribution of released sterile *Ae. aegypti* males. Dengue epidemiologicaldata were also collected from the treated sites to complement the analysis.

## Methods

### Ethics approval statement

The pilot field trial, titled “Field Evaluation of Sterile Insect Technique for *Ae aegypti* Suppression” (NMRR-17–2652-39,099), was funded by a Malaysian National Institutes of Health (NIH) grant. The proposal for the field evaluation was reviewed by the Medical Research Ethics Committee (MREC) and was exempted from MREC review under approval number NMRR-17–2652-39,099 S1 R2.

The SIT using gamma irradiation for dengue vector control, employing a suppression strategy, is not regulated under the Biosafety Act 2007 [Act 678] in Malaysia. The Biosafety Act 2007 primarily regulates the release, importation, exportation, and contained use of living modified organisms (LMO) and the release of products derived from such organisms. Under the Act, LMO refers to any living organism that possesses a novel combination of genetic material obtained through the use of modern biotechnology. Therefore, the SIT approach using gamma irradiation does not fall within the scope of the Act as it does not involve genetically modified organisms.

### Handling and preparation of sterile male pupae of *Ae. aegypti*

One-day-old male pupae of *Ae. aegypti* were obtained from rearing colonies of field strain maintained in the insectary. The male pupae were carefully sex-sorted and irradiated at 55 Gray to ensure they remain sterile upon release; the optimal dosage was determined during the laboratory phase study. These pupae were transferred from a container with a diameter of 13.00 cm, containing seasoned water, to a petri dish layered with white cotton cloth (diameter of 13.50 cm) and filled with approximately 3.0–5.0 ml of seasoned water. Each petri dish was loaded with approximately 4000 pupae by scooping. In one irradiation session, up to 6 petri dishes could be stacked together, accommodating an estimated total of 24,000 pupae. Two dosimeters were attached to the stack of 6 petri dishes for two purposes: one to measure the absorbed dose rate by the pupae at the radius of the petri dish, and the other at the circumference of the petri dish. The mosquito pupae in the petri dishes were enclosed in a metallic container (canister) and were placed in a biosafe gamma radiator, where they were exposed to the relevant dosage of ionizing radiation from Cesium-137. The method was designed and modified with reference to Helinski et al. [26, 30].

The irradiation of male *Ae. aegypti* pupae were conducted in Malaysian Nuclear Agency using Biobeam GM 8000 Irradiator (Manufactured by Gamma Service Medical GmbH Bautzner/ Leipzig/Germany) with Cesium-137 (Cs-137) source. After irradiation at the Malaysian Nuclear Agency, Bangi, the pupae were transferred back to containers with seasoned water and transported to the rearing facility at IMR, 80 km away. Approximately 100 male pupae were scooped into each paper cup, with a minimum of 20–30 ml of water and covered with nets. Pupae mortalities were recorded, and excess water was poured off before each cup containing adult mosquitoes was provided with a 10% sucrose pad for feeding. Pupae mortalities for the entire batch were kept at minimal rate that should not exceed 10% of the total irradiated mosquito pupae. The average room temperature was maintained at 26 ± 3 °C, with an average relative humidity of 72 ± 3%, ensuring optimum rearing conditions in the lab/insectary to maintain the fitness of the mosquitoes, including the irradiated sterile males prepared for field releases. The sterile male mosquitoes, aged between 2 and 4 days old, were then released into the field. The sterile male adult mosquitoes were fed daily with a 10% sugar solution until the release day at the field sites.

### Criteria and selection of treated and untreated sites

**Fig. 1:**
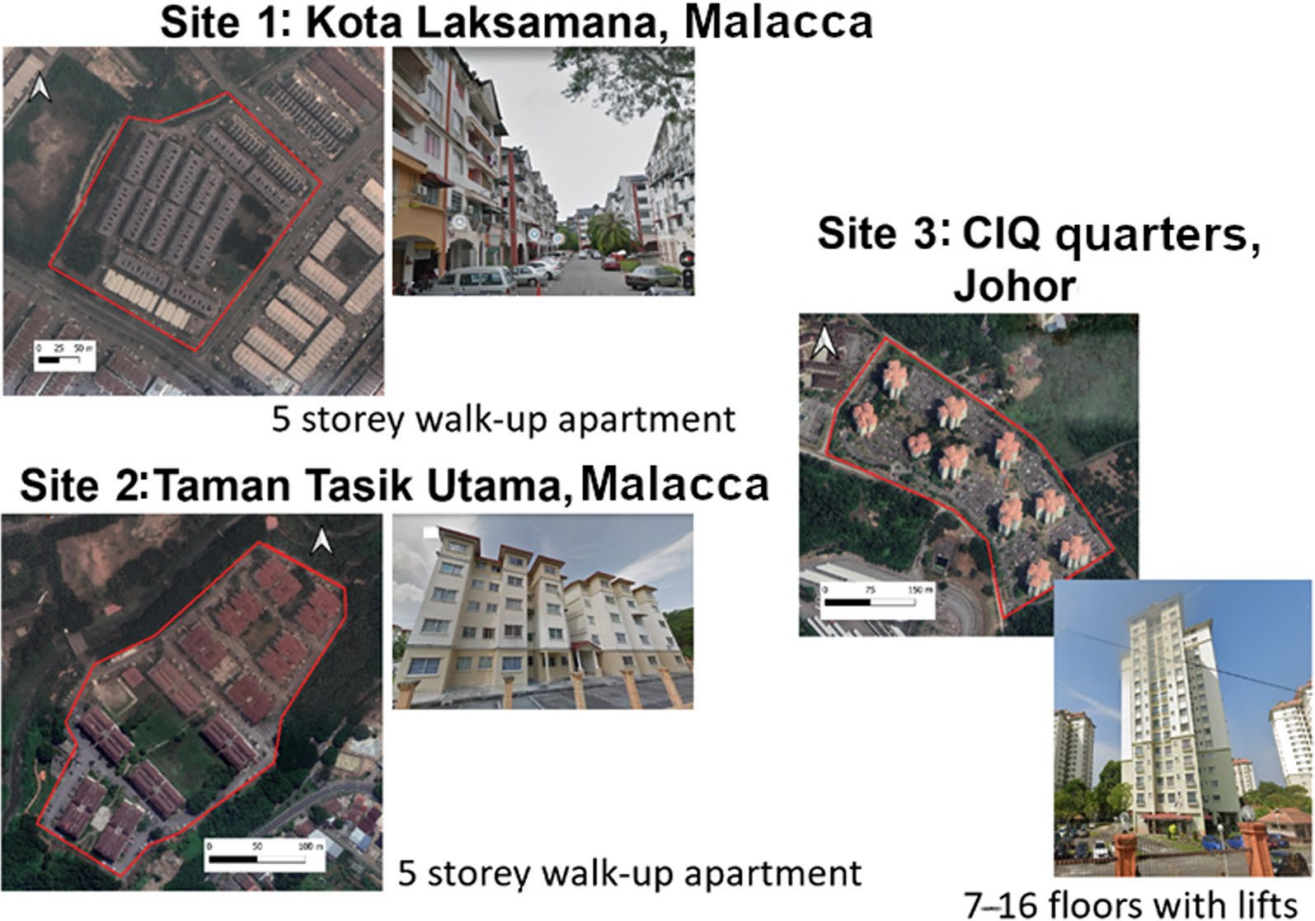
Field treated sites; **a** Site 1: Kota Laksamana, Malacca; **b** Site 2: Taman Tasik Utama, Malacca and **c** Site 3: Quarters for staff of Customs, Immigration and Quarantine Complex, Gelang Patah, Johor

Potential sites were identified based on several criteria such as (i) good community acceptance; (ii) *Ae. aegypti* ovitrap index. The ovitrap index is an entomological measure assessing mosquito populations, focusing on *Aedes* species like *Ae. aegypti* and *Ae. albopictus*, based on egg-laying in ovitraps; (iii) number of dengue cases reported; (iv) Isolated with good natural barriers or boundaries; and (v) easily accessible by road. Two five-storey apartment sites were selected based on the density of *Ae. aegypti* and isolated physical location. The isolated factor is vital to reduce the wild mosquito movement into the treated site from the adjacent area. Initially, Kota Laksamana (KT) 4.55 Ha with 13 residential blocks of four-storey walk-up apartment was selected as treated site while Taman Tasik Utama (TTU) covering 5.95 Ha with 12 residential blocks with 4-storey walk-up apartment was selected as untreated site (Fig. 1a). The release was conducted from 15 July 2019 to 28 December 2020 for 19 months. However, approaching the end of 2020, there was a dengue outbreak at Taman Tasik Utama (Fig. 1b). We could not leave TTU unattended and the Malacca Health Department requested to expand the SIT project in Taman Tasik Utama the outbreak area and switched the untreated site during the trial period. Instead, we released in the untreated site and ceased releasing in the treated site. The KT was then became untreated site and TTU was the treated site.

Prior to release of sterile male *Ae. aegypti* in the trial sites mass fogging and/or mass source reduction including larviding was conducted to reduce the wild *Ae. aegypti* population. Fogging and larvaciding are crucial preparatory and complementary strategies in sterile mosquito release programs [31]. Fogging effectively reduces the adult wild mosquito population, decreasing competition and increasing the penetration of sterile mosquitoes into the release sites. By integrating fogging with larviciding, which targets mosquito larvae in breeding habitats, the intervention creates favorable conditions for release and produces a combined impact, facilitating a long-term reduction in mosquito populations and strengthening vector control programs. Sterilised male *Ae. aegypti* was released at regular weekly basis. The analysis was focused on the comparison of the two periods, the first period where Kota Laksamana was the treated site (year 2019–2020) and Taman Tasik Utama was the untreated site; and the second period where the Kota Laksamana was switched as the untreated site and Taman Tasik Utama became the treated site (year 2021). Releases were also conducted in a highly isolated area in the state of Johor which is in the south of malaysia close to Singapore. The site is the quarters for staff of Customs, Immigration and Quarantine Complex (CIQ), Gelang Patah, Johor (Fig. 1c). This site is 8.41 Ha with 10 residential blocks, a highrise apartment with 7–16 levels.

### Baseline data collection

Prior to releases being initiated, entomological data was collected at each site to estimate population size and to develop appropriate ways of monitoring the mosquitoes. Standard Operating Procedures were developed to monitor the quality of the mosquito colony destined for release during the mass rearing process adapted from Balestrino et al. [32]. Baseline ovitrapping activities were conducted since November 2018 at Pangsapuri Kota Laksamana, Pangsapuri Taman Tasik Utama, Taman Peruna, Taman Indah, Taman Saujana, Seksyen 2, and Taman Bertam Perdana, Bertam Ulu, Malacca. Potential sites were then identified based on the criteria which has been stipulated. Two sites in Malacca were selected based on the ovitrap index of *Ae. aegypti* in both the apartments which were relatively high; ranged from 53–90% for Pangsapuri Kota Laksamana, while 55–62% for Pangsapuri Taman Tasik Utama. In Johor, CIQ quarters was chosen to conduct the the release of sterile male *Ae. aegypti*. The goal of this trial is to suppress the wild *Ae. aegypti* population and curb the transmission of dengue.

### Community engagement

Community engagement is a pre-requisite for any mosquito release programme. Community engagement on SIT was conducted by both Health Education Unit (HEO), Public Health Division, Malacca State Health Department and Johor State Health Department. To engage the public at the release localities were engaged to meet and communicate with local community leaders, provide information on SIT and lead workshops and roadshows. Interactions and meetings were initiated with local government and political leaders. Household surveys were conducted in April to June 2019 to obtain the majority agreement on the release of the sterile *Ae. aegypti* males (SIT). Overall, the residents were supportive towards the release of sterile *Ae. aegypti* males for dengue control.

### Release frequency and quantity of sterile male mosquitoes at three study sites

In the study, sterile male mosquitoes were released at three study sites: KT and TTU in Malacca; and CIQ Quarters in Johor. Each site was characterized as an apartment building type. KT was the first release site in the study, and the release period spanned from 15 July 2019 to 28 December 2020. During this period, a total of 76 releases were conducted, amounting to 2,214,109 sterile males. As the initial release site, KT required substantial troubleshooting and adjustments to establish large-scale mass rearing protocols effectively. Over the first 17 weeks, an average of 15,297 sterile males were released per week. Subsequently, the release rate gradually increased to 21,805 males per week over the following 20 weeks, and ultimately reached an optimal release rate of 38,879 males per week during the final 39 weeks of the study. It is noteworthy that one release was missed due to Movement Control Order (MCO) restrictions during epidemiological week 14 (Ep14/2020). The initial release numbers were intentionally modest to accommodate and stabilize the rearing capacity, which was progressively scaled up from 15,297 to 21,805, before achieving the optimum weekly release rate of 38,879 sterile males. This phased increase in release capacity was essential to overcoming early challenges and ensuring effective population suppression.

In TTU, releases were conducted from 4 January 2021 to 6 September 2021, comprising 32 release events with a total of 1,250,000 sterile males released. The average weekly release rate was approximately 39,062 sterile males. However, two releases were missed due to MCO restrictions during epidemiological week 20 and week 23 of 2021. Additionally, the unavailability of dedicated units for the transportation of mosquitoes for irradiation and delivery to release sites presented logistical challenges. Despite these interruptions, the consistent release strategy was designed to ensure effective coverage and maximize the impact of sterile male dispersal over the study period.

**Fig. 2:**
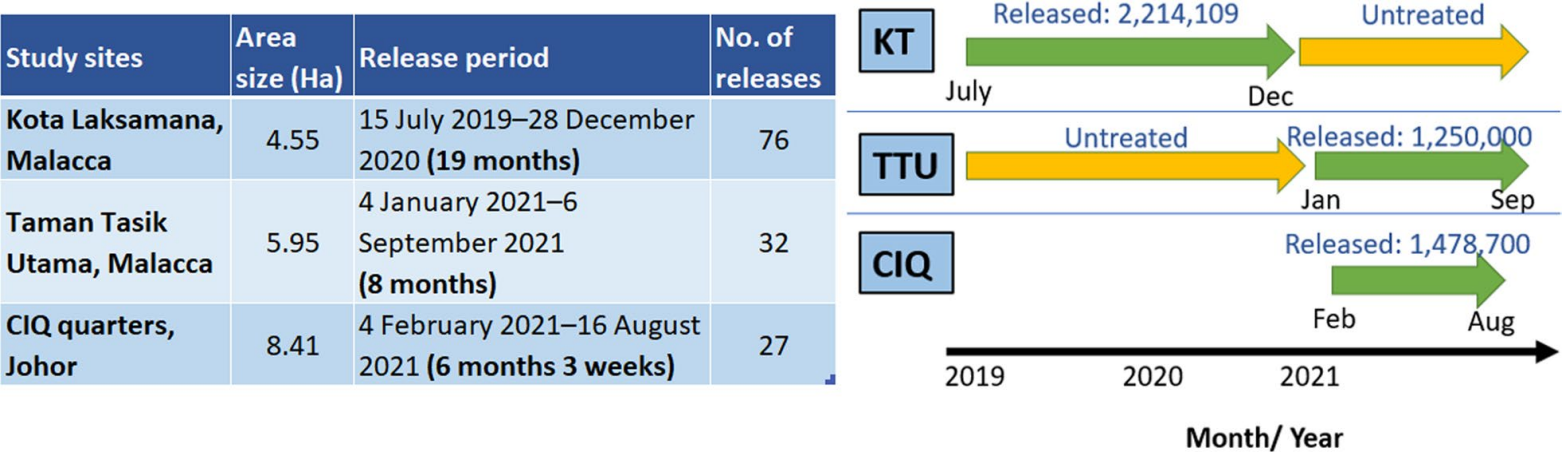
Description of treated sites of field release, and; release frequency and quantity of sterile *Ae. aegypti* male mosquitoes at three study sites in Malacca and Johor. KT: Kota Laksamana; TTU: Taman Tasik Utama; CIQ: Customs, Immigration, and Quarantine Complex (CIQ), Johor

At CIQ Quarters in Johor, 27 releases were conducted between 4 February 2021 and 16 August 2021, with a total of 1,478,700 sterile males released. The average weekly release rate was approximately 54,766 sterile males. Similar to TTU, two releases were missed due to MCO restrictions during epidemiological week 20 and week 23 of 2021 (Fig. 2).

### Mean larval per trap (MLT)

Ovitraps were deployed at the sites for an average of 7–8 days. Upon retrieval, each ovitrap container was transferred to a microwave-safe plastic container, with those containing less than 50 ml of water topped up with seasoned water to optimize hatching conditions. Hatched larvae were reared under controlled conditions to the L3–L4 stages, allowing precise species identification of *Ae. aegypti* and *Ae. albopictus* based on larval morphological characteristics. The ovitrap dataset lacks egg counts specific to the two sympatric species (*Ae. aegypti* and *Ae. albopictus*) in the area, as identifying eggs is challenging due to mixed-species breeding in almost all containers. Only the hatched larvae have been classified and counted by species, therefore it has been possible to perform only the analyses of the percentage of larvae developed from the eggs collected by the ovitraps and the percentage of positive ovitraps.

The larval density was indicated by the mean larval per trap (MLT), the average of the sum of larval per total recovered ovitrap [33]. The lower MLT indicated better suppresion of *Ae. aegypti* population. We studied the MLT for both *Ae. aegypti* and *Ae. albopictus.* A protocol was established in which paddles were removed on the 7th day post-recovery to ensure optimal conditions for larval care. During this period, larvae received an adequate supply of fish food to support healthy development. This regimen allowed precise identification of larvae at the L2–L4 instar and pupal stages after collection, facilitating accurate population estimation while minimizing larval mortality. This approach effectively met larval developmental needs and minimized potential biases in population estimates related to increased larval mortality rates*.*

### Relative variance (RV)

The adequacy and reliability of the monitoring system can be evaluated, according to Service (1993) [34], by measuring the RV, i.e., the ratio between the standard error and the mean number of eggs or adult/trap/week. Southwood and Henderson (2000) defined an RV = 0.25 as usually adequate for most extensive sampling surveys, although in certain intensive programs an RV = 0.1 may be required [35].

### Mark-release-recapture (MRR)

**Fig. 3:**
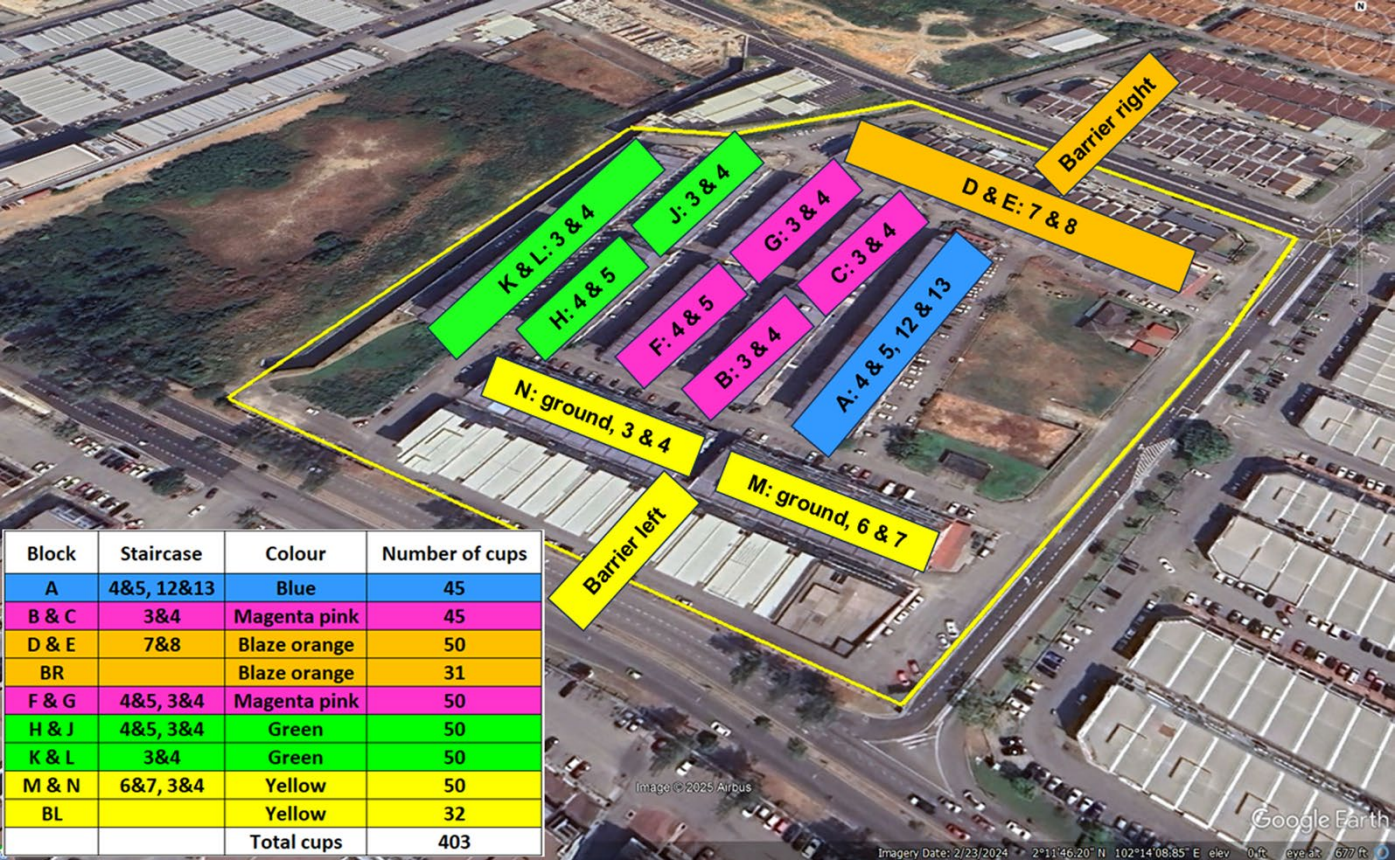
Plan of mark-release-recapture conducted in Kota Laksamana using fluorescent powders of five different colours

The mark-release-recapture was conducted in KT and TTU using 3 days old sterile males. The adult males were marked just before the release, using fluorescent powders of five different colours (green, yellow, blue, magenta and orange) (Fig. 3). Marked males were released in both the SIT treatment areas and the daily recapture were performed by means of 50 sticky traps. The Lincoln Index [36] was used to assess the wild population size of *Ae. aegypti* males and to estimate the sterile:wild males ratio.

### Dengue epidemiolgy data

The dengue cases were obtained from the Health Department of Vector Control Malacca, Ministry of Health Malaysia. The District Health Office's National e-dengue database contains information on every dengue case that has been reported. This epidemiological end point data was obtained from the health department based on the National e-dengue database. Through the use of this National Dengue Surveillance System, dengue cases within the population residing at the research sites were identified. The National Case Definition Guidelines (Case Definitions for Infectious Diseases in Malaysia 2017) were used to diagnose all cases. The use of uniform diagnostic criteria in this system assures that test findings are not biased between SIT treated sites and untreated sites.

### Spatial interpolation of MLT

The spatial interpolations use locational points with known values to estimate values at unknown points in order to create a surface map covering a locational area. In this study, several surface maps of mean larval per trap of *Ae. aegypti* were generated for visual comparison between treated and untreated sites across different period. Only MLT of *Ae. aegypti* above zero was included in the analysis. The spatial interpolation of the mean larval per trap of *Ae. aegypti* was analysed using Ordinary Kriging of ArcGIS (Version 10.6.1, Environmental Systems Research Institute (Esri), Redlands, CA, USA) Geostatistical Wizard. The base maps of the buildings were digitized from the Google Map using QGIS 3.8 (Version 3.8, QGIS, Beaverton, Oregon, USA).

## Results

### Mean larvae per trap

**Fig. 4:**
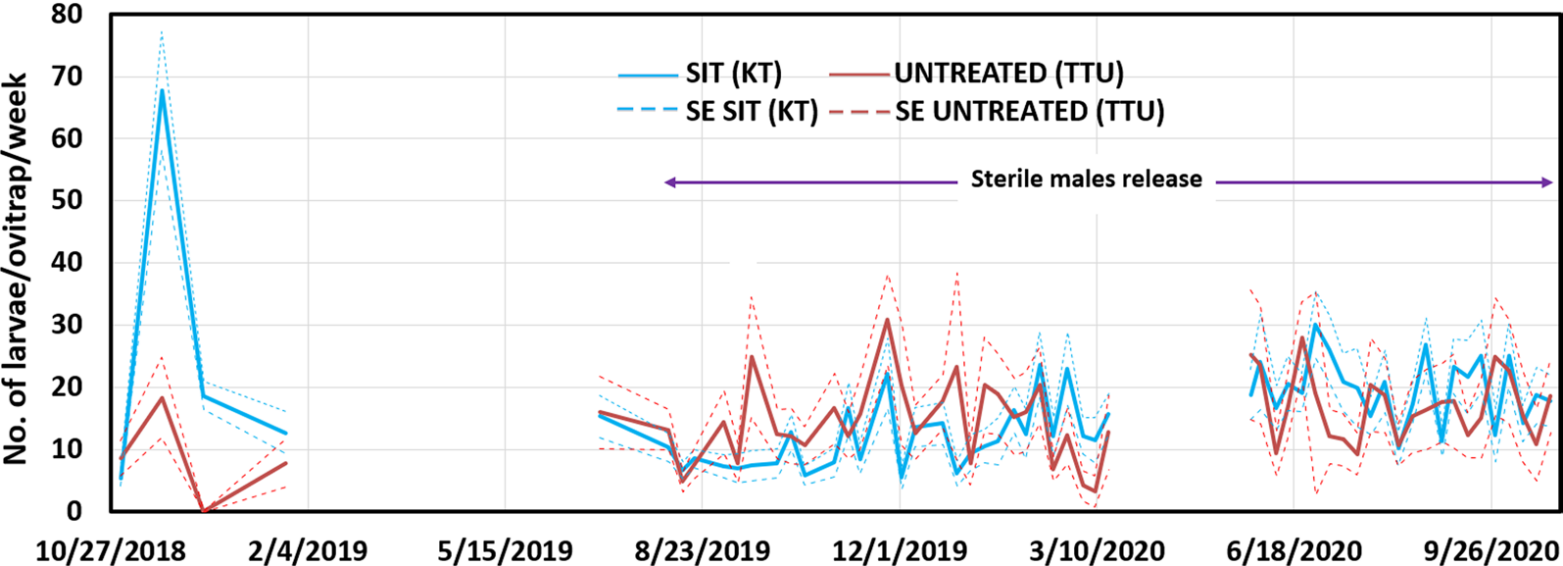
Number of *Ae. aegypti* larvae hatched from eggs collected in the SIT treated area (KT) in comparison to the untreated area (TTU) (mean ± *se*). KT: Kota Laksamana; TTU: Taman Tasik Utama

**Fig. 5:**
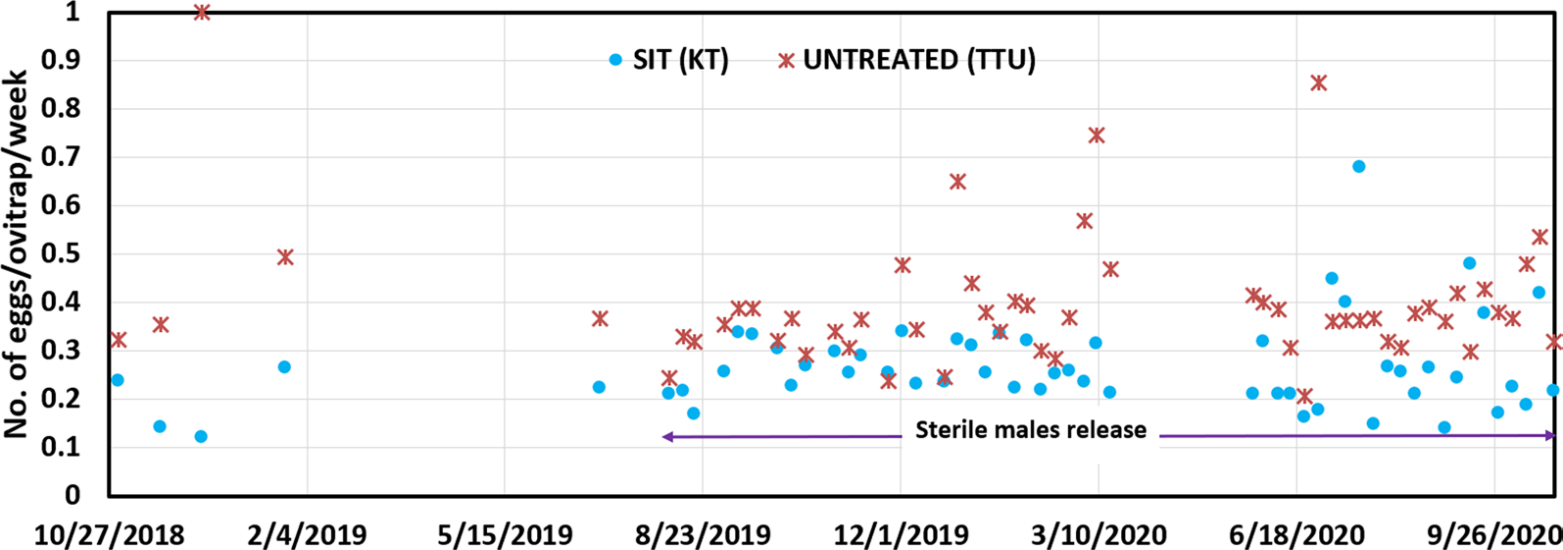
The relative variation of number of larvae hatched from eggs/ovitrap/week in SIT treated (KT) and untreated (TTU) areas. RV: Relative variance; KT: Kota Laksamana; TTU: Taman Tasik Utama

The analysis of ovitrap data (Fig. 4) does not show significant differences (*P* = 0.940) between the number of *Ae. aegypti* larvae developed from the eggs collected in the treatment area (15.37 ± 6.52 larvae/ovitrap per week) and in the untreated area (15.46 ± 6.15 larvae/ovitrap per week) during the releases. The descriptive data from the graph indicates a difference in the number of larvae per trap per week between treated (KT) and untreated (TTU) sites, as illustrated in Fig. 4.Baseline data prior to the release show that KT had an average of nearly 70 *Ae. aegypti* larvae per trap per week. An independent sample *t*-test was conducted to compare the mean larval per trap of *Ae. aegypti* per trap between the baseline periods. The results showed no significant difference between the two baseline periods of 2 sites, *P* = 0.321. In the TTU site, which served as the untreated control, exhibited approximately 25 larvae per trap per week prior to the release. During the same timeframe as the KT release period, TTU larvae counts fluctuated, reaching a maximum of around 30 larvae per ovitrap per week (*P* = 0.062). The Relative Variation (RV = SE/mean–measures the adequacy of monitoring) in SIT treated area is close to 0.30 (RV = 0.25 is usually considered adequate for most extensive sampling surveys) while in the untreated area RV is significantly higher than 0.30 indicating that an insufficient number of ovitraps were activated (Fig. 5).

**Fig. 6:**
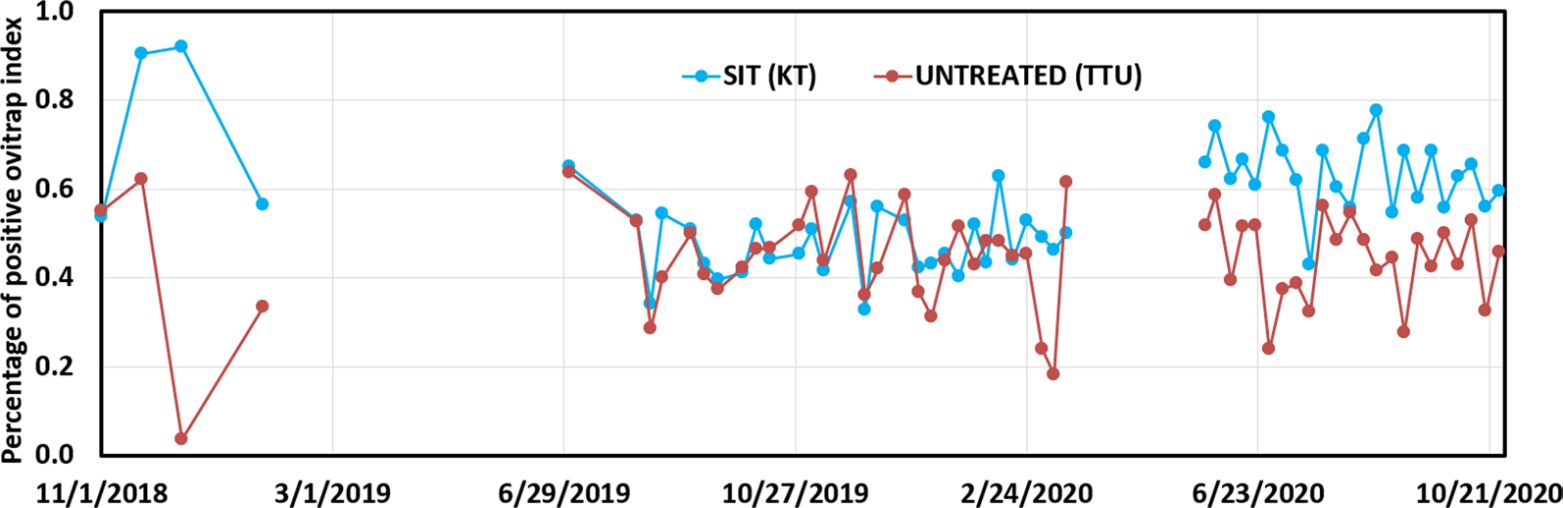
Percentage of positive ovitrap index in SIT treated and untreated areas. SIT: Sterile insect technique

The percentage of positive ovitrap index (OI) in the SIT treated area (OI = 0.54 ± 0.50) during the sterile male releases is significantly higher (*t*-test for independent samples *P* < 0.01) than in the untreated area (OI = 0.44 ± 0.50) (Fig. 6).

**Fig. 7:**
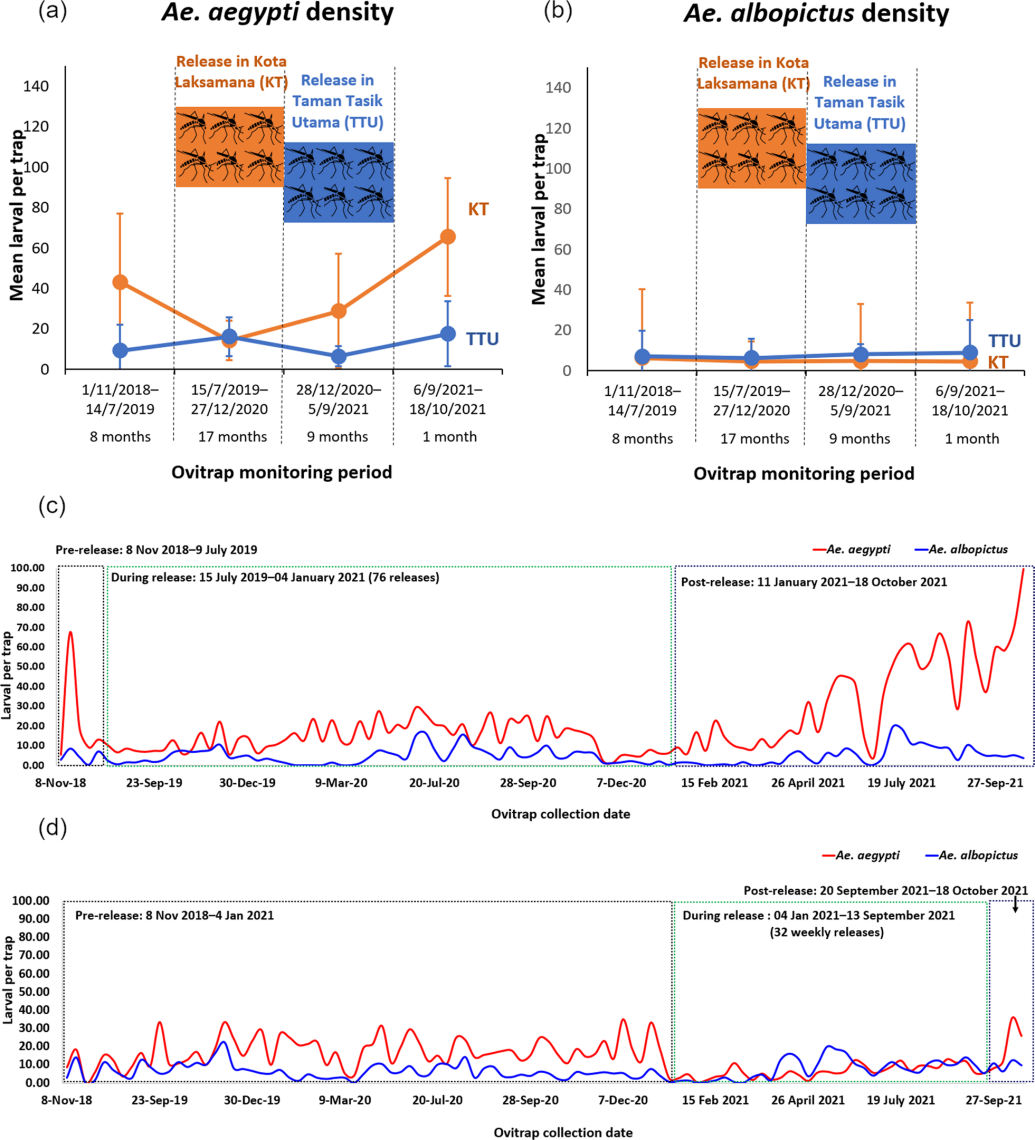
**a** Mean larval per trap of *Ae. aegypti* density; **b** Mean larval per trap of *Ae. albopictus* density in KT and TTU, Malacca; **c** The mean larval per trap for *Ae. aegypti* and *Ae. albopictus* during the pre-release, during release and post release periods in KT; **d** The mean larval per trap for *Ae. aegypti* and *Ae. albopictus* during the pre-release, during release and post release periods in TTU. KT: Kota Laksamana; TTU: Taman Tasik Utama

A total of 76 releases were conducted in KT, starting with 16,500 sterile males for the initial release and increasing to an average of 38,000 sterile males from the 36th release onwards. Prior to the release period, the mean larval per trap of *Ae. aegypti* in KT doubled the MLT in TTU. In KT, during the release of sterile male *Ae. aegypti*, the mean larval per trap of *Ae. aegypti* was reduced to the lowest (Fig. 7a). The MLT of *Ae. albopictus* in KT and TTU did not vary much throughout the study period (Fig. 7b). KT achieved 76.25% suppression of *Ae. aegypti* larvae compared to TTU; the untreated site after 76th releases. *Ae. aegypti* larval density at KT showed significantly lower density post 76 releases compare to baseline data (*t*-test; *P* < 0.0001). However*, Ae. aegypti* larval density at the untreated site; TTU indicated no significant difference between pre and during-release phase (*t*-test; *P* = 0.263). When releases were stopped, the mean larval density in KT rebounded to levels as high as those seen in the pre-release period after 14 weeks (3.5 months); and within 6 weeks of the post-release phase. During the release phase, *Ae. aegypti* decreased by 1.6 times compared to pre-release levels, with MLT decreasing from 23 to 14, while *Ae. albopictus* larval density experienced a slight increase of onefold. It was interesting to observe that during one of the during-release monitoring, the *Ae. aegypti* MLT has dropped to a level of 2 larvae per trap. The *Ae. aegypti* MLT rose to 20 larvae per trap in average in post-release phase (Fig. 7c). The *Ae. albopictus* to *Ae. aegypti* ratio was 1:7 during the post-release period. On the other hand, in TTU, the release site, the mean larval count of *Ae. aegypti* per trap was reduced to its lowest level during the release period from December 2020 to September 2021. TTU achieved a 96.74% suppression of *Ae. aegypti* larvae compared to KT, the untreated site, after the 16th release. Five weeks post-release phase at TTU, larval density rose to 20 larvae per trap, this finding was similar to post-release monitoring in KT (Fig. 7d). The ratio of *Ae. albopictus* to *Ae. aegypti* was 1:2 during the post-release period.

**Fig. 8:**
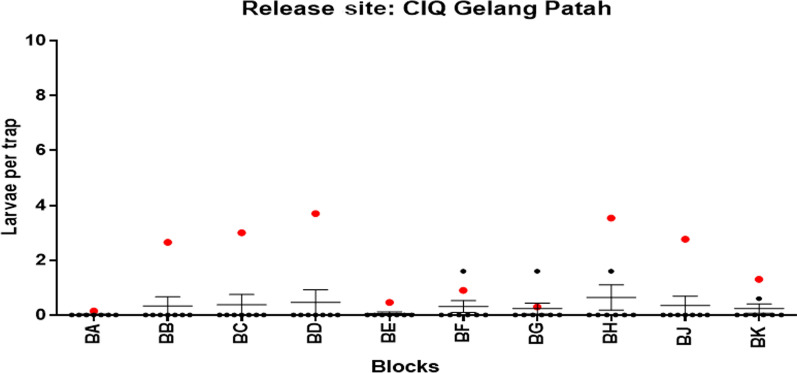
Comparison of mean larva per trap by blocks and the baseline data at Customs, Immigration, and Quarantine Complex (CIQ), Johor

In CIQ Gelang Patah, the 3rd release site, a total of 16 releases with 874,500 sterile males were performed on a weekly basis from 4th February 2021 to 24th May 2021 (3.5 months). The suppression of larval density per trap was 89.00% compared to the average baseline data of the CIQ, Gelang Patah. Most of the blocks had 0% *Ae. aegypti* indicating 100.00% suppression (Fig. 8).

### Mark-release-recapture (MRR)

**Fig. 9:**
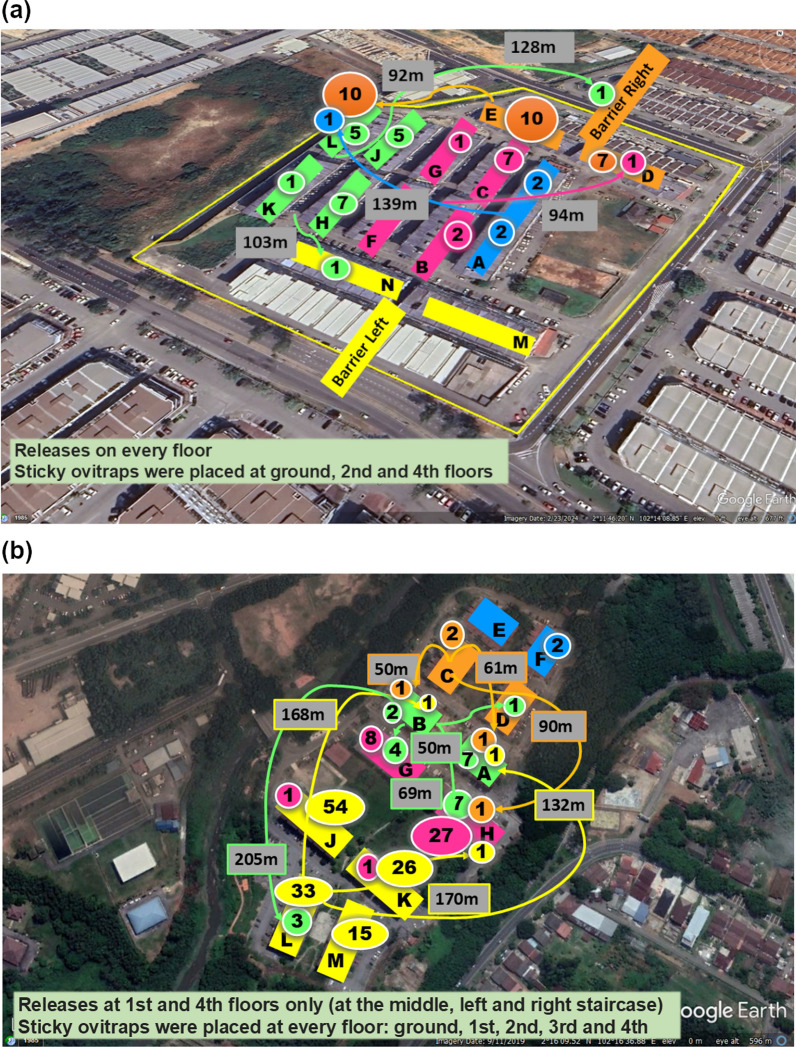
Mark-release-recapture of sterile males conducted at **a** Kota Laksamana and **b** Taman Tasik Utama

The daily survival rate in the MRR trials was from 0.54 to 0.71 and the mean daily survival rate (± *SD*) was 0.61 (± 0.08). The rate sterile/wild males ranged from 13:1 in the first day after release to 1:1.5 on the seventh day. The results showed that *Ae. aegypti* sterile male released weekly at the dose range of 1278–7942 males/ha per week, corresponding to a mean ratio of sterile/wild males of 5.85 (range: 0.59–17.04) (Fig. 9).

### Dengue epidemiology data

The dengue cases were reduced during the release of sterile male *Ae. aegypti* for both sites. During the release period in KT, only 7 dengue cases were reported, indicating a low incidence of dengue transmission. After the final release on December 28, 2020, post-release monitoring was initiated to assess any delayed impact on dengue incidence. The first post-release dengue case appeared 16 weeks after the last release, and over the subsequent 37 weeks, an additional 6 dengue cases were recorded. A slight increase in dengue cases was observed during this post-release phase, with a peak of 3 cases occurring within a single week. This trend suggests that the SIT intervention contributed to a reduction in dengue cases during the release period, with a gradual rise in cases following the cessation of releases.

**Fig. 10:**
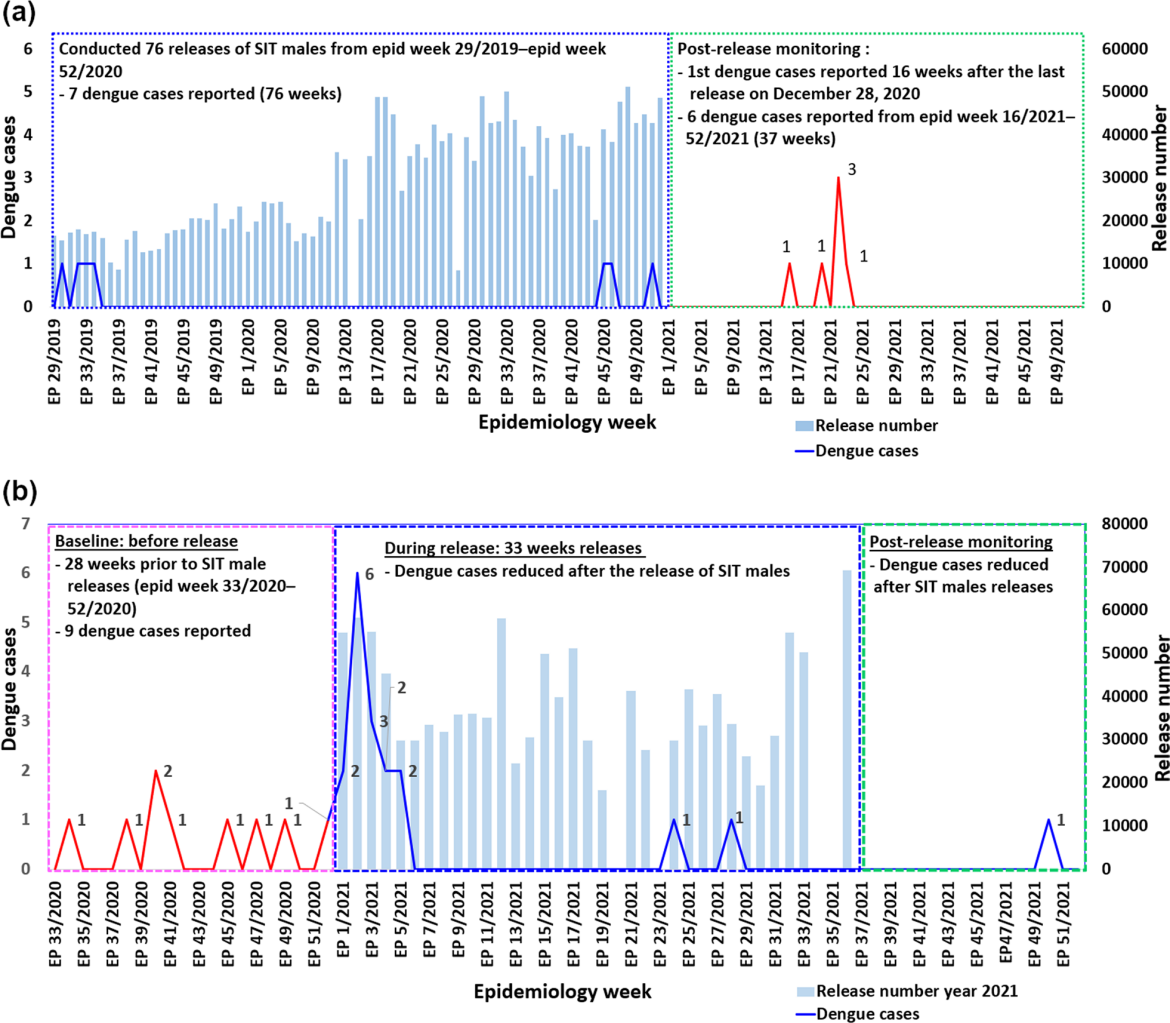
Dengue cases at **a** Kota Laksamana and **b** Taman Tasik Utama

In Taman Tasik Utama (Fig. 10a), the dengue virus transmission was controlled after releasing of sterile male *Ae. aegypti* (Fig. 10b). During the 28-week baseline period prior to the release of sterile male mosquitoes (SIT), a total of 9 dengue cases were reported. Over the subsequent 33-week release phase in 2021, dengue cases initially increased but then declined markedly, with several weeks showing zero reported cases. The reduction of dengue cases was observed after five weeks of field release (Fig. 10b). Post-release monitoring revealed a sustained reduction in dengue incidence, with only one case reported 14 weeks after the release had ceased.

### Spatial characteristics of the released *Ae. aegypti* males

**Fig. 11:**
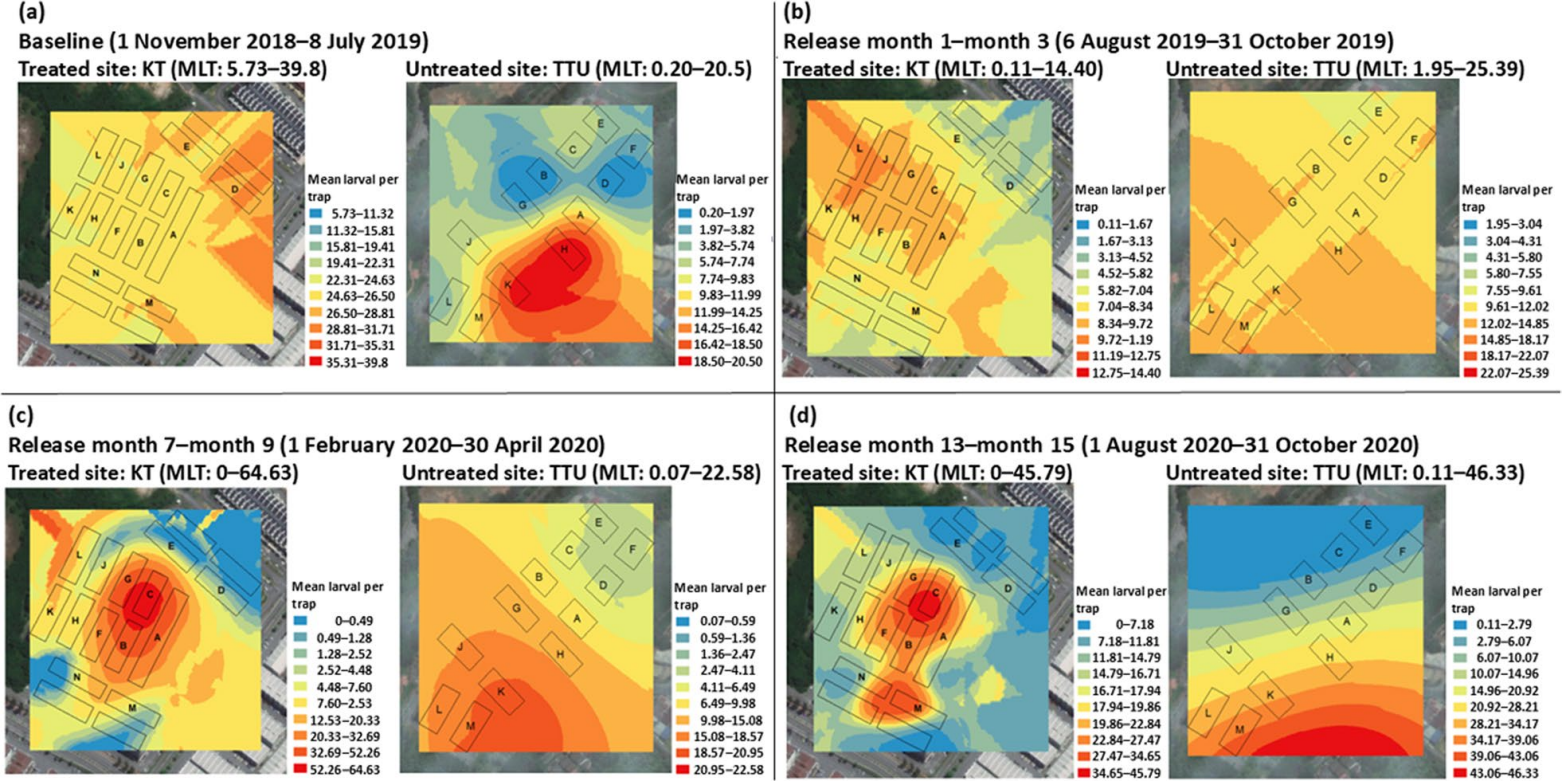
Spatial clustering of the mean larval per trap of *Ae. aegypti* in KT (treated site) and TTU (untreated site) during **a** baseline period; **b** release first to third month; **c** release seventh to ninth month; **d** release thirteenth to fiftieth. KT: Kota Laksamana; TTU: Taman Tasik Utama; MLT: mean larval per trap

From 2018 to 2020, KT was the treated site while TTU was the untreated site. During the initial release period for the first three months, there was no spatial clustering of the mean larval per trap of *Ae. aegypti* in the treated site (Fig. 11a). The mean larval per trap of *Ae. aegypti* was clustered at the center of the KT with the maximum MLT of 64 from the month 7 to 9 of release (Fig. 11c) and maximum MLT of 45 from the month 13 to 15 (Fig. 11d).

**Fig. 12:**
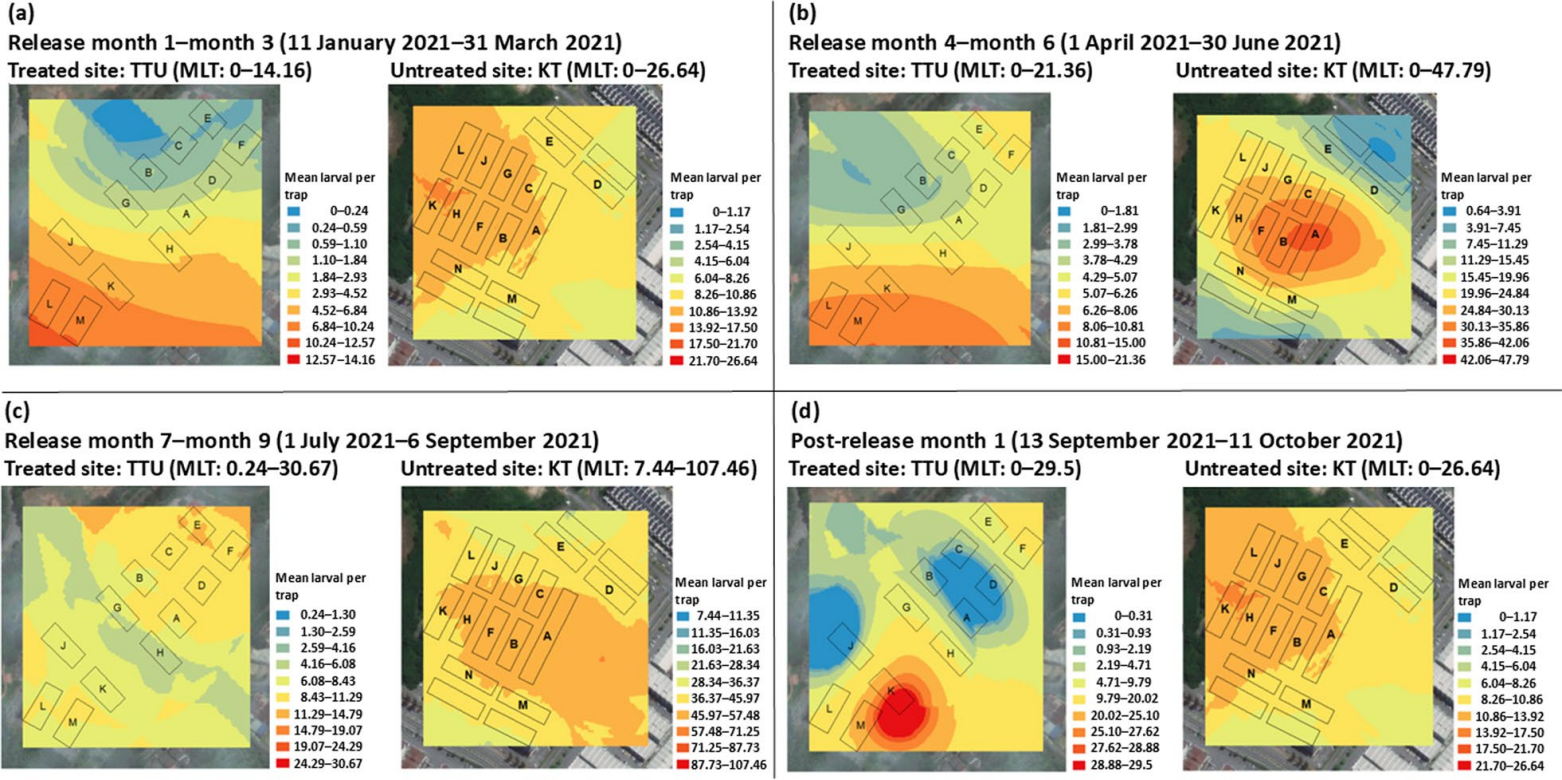
Spatial clustering of the mean larval per trap of *Ae. aegypti* in TTU (treated site) and KT (untreated site) **a** release first to third month; **b** release fourth to sixth month; **c** release seventh to ninth month; **d** post-release first month. KT: Kota Laksamana; TTU: Taman Tasik Utama; MLT: mean larval per trap

In 2021, TTU was the treated site while KT was switched as the untreated site. During the release period of nine months, no spatial clustering of the mean larval per trap of *Ae. aegypti* was observed at the treated site (Fig. 12a). The mean larval per trap of *Ae. aegypti* was clustered around the block K and block M at the maximum of 29 during the post-release period (Fig. 12d). The untreated site showed no spatial clustering throughout the study period. Overall, the mean larval per trap of *Ae. aegypti* at Taman Tasik Utama was relatively low compared to Kota Laksamana during release period.

## Discussion

The results of this study highlight a notable change in larval abundance during the release period in KT compared to baseline conditions. These findings emphasize the effectiveness of Sterile Insect Technique male releases in reducing the *Ae. aegypti* population, ultimately contributing to a reduction in dengue transmission. The study was conducted in two phases. The first phase involved laboratory experiments to assess the bionomics of local *Aedes* mosquitoes and to determine the optimal sterilization dosage for the local *Ae. aegypti* strain [37]. These experiments identified the optimal irradiation dosage as 55 Gy. The second phase consisted of open field releases of sterile male *Ae. aegypti* mosquitoes, and the effectiveness of this approach was evaluated through entomological surveillance, including the ovitrap index and dengue epidemiological data provided by the Malacca and Johor State Health Departments. In all release localities, counting eggs was challenging due to the presence of both *Ae. aegypti* and *Ae. albopictus* in the same containers. In Malaysia, *Ae. aegypti* and *Ae. albopictus* are the primary vectors for dengue virus transmission. Extensive habitat monitoring studies in Malaysia, Singapore, and Thailand, all with similar climates, have shown that *Ae. aegypti* breeds equally in both indoor and outdoor artificial containers, while *Ae. albopictus* is the dominant breeder in outdoor containers, outnumbering *Ae. aegypti* by 2.34–fold. This adaptation of both vectors to human environments underscores the need for targeted vector control strategies that address the unique ecological and behavioral traits of these species [38].

Identifying the abundance of eggs of both species*, Ae. aegypti* and *Ae. albopictus*, proved challenging under microscope. Therefore, we adopted an approach focused on identifying egg hatch and counting the larvae of both species.

The SIT deployments in KT, TTU, and CIQ sites effectively reduced *Ae. aegypti* larval densities, achieving suppression rates of 76.25%, 96.74%, and 89.00%, respectively. In KT, sustained weekly releases over 18 months led to consistent suppression in an urban area. At TTU, a shorter release period resulted in near-total suppression, demonstrating SIT’s effectiveness in high-density residential settings. In the isolated CIQ site in Johor, substantial reductions were observed, highlighting SIT’s success in contained environments. During the releases at a point there was on-going adjacent new construction site and the invasion of wild mosquities at the CIQ treated site, hence mean larvae per trap started to built up.

The MRR study showed that total number of mosquitoes to be release was sufficient for the treated sites. We released 13 sterile mosquitoes per wild mosquito on the first day of release and reduced to 1:1.5 at the seventh day after release. This indicates that there is invasion by wild *Ae. aegypti* into the release area as shown by MRR study. This could also be the reason of not achieving > 90% suppression due to the invasion of mosquitoes from adjacent area. Kota Laksamana is located in the town with no natural boundaries and many amenities. The SIT trial area was not sufficiently isolated and migration of already mated females from outside the SIT area, would have played a significant role. Since the mean survival rate (± *SD*) was 0.61 (± 0.08), instead of releasing the sterile mosquitoes on a weekly basis we could have released two times a week which may provide a better suppression data in the study sites. The MRR trials provided added information on data of how far the coloured male mosquitoes could fly. In KT, the mosquitoes could move between blocks and the farthest distance was 139 m. However in TTU, the mosquitoes movement between blocks were ever further to 205 m.

Overall analysis of releases showed that in SIT treated area the OI is higher by about 11% than untreated, indicating higher number of eggs [39] observed that the percentage of positive ovitraps was correlated to population density) while the *Ae. aegypti* larvae hatching by eggs collected in SIT treated area is 14% lower than untreated, these two data show that the releases of sterile males induced sterility.Spatial clustering of *Ae. aegypti* was observed at the 7th—9th month of release and 13th –15th month of release in KT. The *Ae. aegypti* clustered at central blocks when the overall mean larval per trap was higher. We released fixed number of SIT male *Ae. aeygpti* throughout the study. The timely kriging analysis on the dispersal and concentration of the *Ae. aegypti* might help in the release of SIT males. In this case, we could focus the release of more steriled males in the central blocks to suppressthe population as the insect population are not stable [40] and the release area is small.

Despite all these, there was a reduction of dengue cases during the period of release in both the treated sites. In particular, during the switching of the treated and untreated sites, the dengue cases in TTU was successfully reduced and controlled after 6 weeks of release. The interaction between SIT male number and dengue cases is complex, as it involves factors such as human mobility, virus transmission, virus generation time, absence/presence of wild population, and improved patient care and control measures.

By suppressing the population of *Ae. aegypti*, SIT targets the primary vector of dengue transmission, effectively reducing mosquito density and lowering the risk of disease spread. Unlike chemical control methods, SIT does not rely on insecticides, which minimizes environmental impact and reduces the risk of resistance development, making it crucial for long-term dengue management. The economic benefits of SIT are also notable, as reducing dengue cases can result in cost savings for the healthcare system and communities, while simultaneously improving public health outcomes.

Overall, the ongoing expansion of SIT in Malaysia highlights its potential to transform the dengue control landscape and enhance public health resilience. In areas treated with SIT, a reduction in *Ae. aegypti* populations could lead to fewer mosquitoes capable of transmitting the virus. However, comprehensive field trials with extended-release periods and larger release areas are necessary to clearly demonstrate the impact of SIT on dengue epidemiology. Well-designed studies and robust analytical approaches are crucial for understanding the effects of SIT on *Ae. aegypti* populations and dengue incidence, ultimately guiding evidence-based control strategies.

Limitations included an insufficient amount of baseline data; ideally, at least six months of baseline data is needed to accurately reflect real-time population sizes of *Ae. aegypti* and *Ae. albopictus* mosquitoes. The challenge of distinguishing *Ae. aegypti* and *Ae. albopictus* at the egg stage, which required labor-intensive rearing for accurate identification and may have been affected by cannibalism. The initial release site, KT, was located in proximity to numerous residential areas, which likely facilitated the influx of *Ae. aegypti* from these surroundings. Additionally, the SIT trial area lacked adequate isolation, allowing for the immigration of already mated females from outside the designated area, which may have significantly influenced the outcomes of the trial. The Lincoln Index estimated the wild male population, with sterile-to-wild ratios decreasing from 13:1 to 1:1.5 within a week, averaging 4.62. The findings suggest biweekly releases in divided quantities could enhance mating between sterile males and wild females, underscoring the importance of maintaining adequate release-to-wild ratios and optimizing spatial distribution. These findings highlight the importance of achieving an adequate ratio of released males to wild females for the successful implementation of a suppression trial using the SIT. The observed sterile/wild male ratio appears to be influenced by spatial distribution; therefore, sterile males need to disperse from treated sites to reach natural courtship and mating areas where wild males and females are present. Implementing methods by targetting release based on the spatial clustering would improve the suppression performance to ensure a more homogeneous distribution of sterile males may significantly benefit the SIT program.

Future efforts should focus on optimizing release strategies and enhancing monitoring techniques to improve the efficacy of vector control interventions.Further works on development of genetic sexing strains (GSS) is also needed to further improve the applicability of Sterile Insect Technique (SIT) / Inherited Sterility (IS) [41], mathematical modelling and dynamic spatial interpolation analysis. Stable SIT suppression result is anticipated if release is conducted at larger area according to the next phase of phased conditional approach [28].

## Conclusions

The study found that releasing sterile male *Ae. aegypti* mosquitoes using the Sterile Insect Technique significantly reduced mosquito populations in various locations. The method achieved a high sterile-to-wild male ratio and a strong daily survival rate. As a result, larval density was notably reduced across different areas, demonstrating the effectiveness of SIT in controlling dengue transmission.

Mark-release-recapture trials confirmed a stable sterile-to-wild male ratio, especially in TTU, though larval densities rebounded after releases stopped, indicating a need for sustained or optimized release schedules. Spatial analysis showed no initial clustering, but patterns emerged over time in KT, suggesting that, *Ae. aegypti* may migrate from nearby areas, highlighting the need for site isolation. These insights provide a foundation for scaling up field releases, refining monitoring, and achieving sustainable vector control with SIT. Future efforts should focus on even distribution of sterile males to ensure they reach natural courtship areas and refine release and monitoring strategies for effective suppression.

## Data Availability

The data that support the findings of this study are available from Nazni Wasi Ahmad but restrictions apply to the availability of these data, which were used under license for the current study, and so are not publicly available. Data are however available from the authors upon reasonable request and with permission of Nazni Wasi Ahmad.

## Abbreviations

AW-A: Areawide application
Bti: *Bacillus thuringiensis israelensis*
CIQ: Customs, Immigration and Quarantine Complex
Cs-137: Cesium-137
Ep: Epidemiological week
GSS: Genetic sexing strains
HEO: Health Education Unit
IS: Inherited Sterility
KT: Pangsapuri Kota Laksamana
LMO: Living modified organisms
MCO: Movement Control Order
MLT: Mean larval per trap
MREC: Medical Research Ethics Committee
MRR: Mark-release-recapture
NIH: National Institutes of Health
OI: Ovitrap index
RV: Relative variance
SIT: Sterile Insect Technique
TTU: Pangsapuri Taman Tasik Utama
